# Mind the gap: an analysis of core capacities of the international health regulations (2005) to respond to outbreaks in Yemen

**DOI:** 10.1186/s12913-021-06395-3

**Published:** 2021-05-20

**Authors:** Hanan Noman, Fekri Dureab, Iman Ahmed, Abdulwahed Al Serouri, Taha Hussein, Albrecht Jahn

**Affiliations:** 1 Heidelberg Institute of Global Health, Uniklinikum, Heidelberg, Germany; 2 Ruprecht-Karls-Universität Heidelberg, Heidelberg, Germany; 3 IRIA, Akkon-Hochschule für Humanwissenschaften, Berlin, Germany; 4 Independent Global Health Expert, Montreal, Canada; 5 Yemen Field Epidemiology Training Program, Ministry of Public Health and Population, Sana’a, Yemen; 6 Medicines Sans Frontiers –France, Aden, Yemen

**Keywords:** International health regulation, COVID-19, Conflict, Yemen

## Abstract

**Background:**

Yemen that has been devastated by war is facing various challenges to respond to the recent potential outbreaks and other public health emergencies due to lack of proper strategies and regulations, which are essential to public health security. The aim of this study is to assess the implementation of the International Health Regulations (IHR 2005) core capacities under the current ongoing conflict in Yemen.

**Methods:**

The study simulated the World Health Organization (WHO) Joint External Evaluation (JEE) tool to assess the IHR core capacities in Yemen. Qualitative research methods were used, including desk reviews, in-depth interviews with key informants and analysis of the pooled data.

**Result:**

Based on the assessment of the three main functions of the IHR framework (*prevention, detection, and response*), Yemen showed a demonstrated or developed capacity to detect outbreaks, but nevertheless limited or no capacity to prevent and respond to outbreaks.

**Conclusion:**

This study shows that there has been poor implementation of IHR in Yemen. Therefore, urgent interventions are needed to strengthen the implementation of the IHR core capacities in Yemen. The study recommends 1) raising awareness among national and international health staff on the importance of IHR; 2) improving alignment of INGO programs with government health programs and aligning both towards better implementation of the IHR; 3) improving programmatic coordination, planning and implementation among health stakeholders; 4) increasing funding of the global health security agenda at country level; 5) using innovative approaches to analyze and address gaps in the disrupted health system, and; 6) addressing the root cause of the collapse of the health services and overall health system in Yemen by ending the protracted conflict situation.

**Supplementary Information:**

The online version contains supplementary material available at 10.1186/s12913-021-06395-3.

## Background

Yemen, which has been devastated by war, famine, a weak health system and overall alarming basic health and nutrition indicators, is facing various challenges in its continued efforts to respond to outbreaks and other public health emergencies. The major reason for that is the lack of a proper health security strategy and regulations to ensure effective implementation, which are essential to public health security. Yemen has lived under the turmoil of several rounds of internal armed conflict and political instability since 2011. The overall impact of war and conflict on the health system is vividly observable.

The International Health Regulations are a legally-binding instrument of international law for the world’s 196 countries including the 194 WHO Member States [1]. The Global Health Security Agenda describes the proactive and reactive measures needed to protect the global population against acute public health threats [2, 3]. In today’s increasingly interconnected world, local public health threats can quickly transform into a Public Health Emergency of International Concern (PHEIC), which in turn can directly impact human lives as well as economic and political stability [2].

Currently, several Low Income Countries (LICs) are unable to implement the International Health Regulations (IHR 2005) core capacities [3]. This is despite the prime purpose of the IHR 2005 itself in ensuring capacity building at national level to achieve the collective goal of global health security [2, 4]. The core capacities impacted include human resources, surveillance, laboratory, response, legislation, policy and financing, coordination, advocacy and national focal point communications, preparedness, and risk communication [5].

The revised IHR (2005) further instructs governments on the modalities of participating in global health surveillance networks through reviewing their national surveillance strategies and implementing practical and relevant response programs that enable the concerned states to contribute to the global health security agenda [6]. This is essential for the response to the twenty-first century’s international public health challenges including epidemic-prone diseases of global concern such as cholera, plague, yellow fever and most recently COVID-19 [5]. Multiple, poorly integrated, donor-driven surveillance systems often spring up in situations of war, totally neglecting the destroyed health systems and focusing funds and efforts on surveillance, thereby titling the balance towards better detection, yet poor capacity to prevent or respond to such outbreaks [6].

A major goal of implementation of the IHR (2005) framework is to bolster countries’ national public health system through the inclusion of an activated disease surveillance system for public health emergencies, however many countries fail to achieve this goal [4]. To ensure the effective application of the IHR core capacities, states can participate in a voluntary tool called the World Health Organization Joint External Evaluation (JEE), which is used for monitoring and evaluating the countries’ capacity to prevent, detect, and respond to the infectious diseases [7].

In Yemen, nearly 102,000 people have died since the beginning of the ongoing war, as a direct effect to the war, and 131,000 from indirect effects including the severe lack of essential and vital services like food, health and shelter according to the United Nations [8]. The ongoing war that started in 2011 has affected different sectors of life throughout the country, including the social, economic, health, humanitarian, and educational sectors [9]. The direct impact of the breakdown of these essential services on the population has resulted in massive human suffering from the lack of basic needs including essential health services [9].

One of the outcomes of the ongoing conflict is the serious deterioration of the already crippled health system in Yemen. Approximately 2.6 million of children under 15 years old are threatened by measles [10]. Furthermore, almost 1.8 million children are at risk of malnutrition [11]. There is a rise in communicable diseases, such as malaria, HIV, and tuberculosis which results in many deaths that could have been treated or easily prevented [12]. The already fragile health system was further stressed by a cholera outbreak as well as various types of communicable diseases such as COVID-19 which has resulted in an increasingly difficult work environment and an even heavier work-load for mostly unpaid healthcare personnel [13].

The challenges to respond to recent potential outbreaks and other public health emergencies in Yemen are primarily due to lack of proper strategies and regulations, which are essential to public health security. Therefore, the present study was vital to assess the status of implementation of the IHR (2005) and the ability of the country to address public health emergencies under the current ongoing conflict and several outbreaks in Yemen.

## Methods

### Study design

This study used a combination of qualitative research methodologies to assess the implementation of the IHR (2005) in Yemen by analyzing the country’s scores obtained through simulating the Joint External Evaluation (JEE) of the IHR (2005) core capacities Yemen [14], along with data obtained through semi structured key informant interviews with public health leaders from the Government, local Non-Governmental Organization (LNGOs) and International Non-Governmental Organizations (INGOs) in Yemen.

### Sampling

Purposive sampling was used to select key informants, who are public health personnel instrumental in implementing IHR (2005) in Yemen, to be interviewed for the study. A total of seventeen key public health informants were invited to participate via email from the following institutions: Ministry of Public Health and Population (MoPHP) (four), International Organizations (seven), independent consultants (two), the National Central Public Health Laboratories (NCPHL) (two), and Academia (two).

### Data collection

In depth interviews (IDI) were conducted with only ten (10) out of the seventeen (17) key health informants who were invited. The other seven (7) key informants did not respond due to political reasons or could not be reached. The Selection of the key health informants was based on their position in relation to implementing IHR (2005) in Yemen. Initially, an information sheet and consent form of the study were sent to all key informants to participate in the interview. After obtaining key informants’ consent to participate in this study, the interviews were conducted via Skype and recorded. Researchers utilized an IDI guide that was developed specifically for this study (see supplementary file). Four (4) out of the ten (10) key informant interviews were conducted in English, with the response notes being transcribed in English as well, while the other six (6) were conducted in Arabic, and the response notes were transcribed in Arabic, then translated to English. Hence, the data was rendered in English for further analysis. The interviews audio recordings and notes were securely saved.

The researcher identified and reviewed IHR (2005) related documents from MoPHP in Yemen, based on the following search keywords:

“IHR”, “Health Stakeholders”, “National health Legislations”, “IHR coordination, communication and advocacy”, “Quarantines”, “Radiological Emergencies”, Chemical Emergencies“, Points of entries”, “Risk Communication”, Emergency Response Operations”. Data from the MoPHP documents was coupled with the data from key health informant interviews -who have vast field experience in Yemen- to complete the scoring exercise of all the IHR (2005) core capacities for Yemen.

### Data analysis

All the ten (10) interviews were transcribed verbatim by a member of the research team. Each interview was given a special secure number (from one to ten) for anonymity purposes.

The ten interview transcripts that were then available in English (after translation of the six interviews conducted in Arabic) were analyzed manually using qualitative content analysis [15]. Four data analysis steps were followed, in order to gain insight into the key informants’ perceptions of the implementation of the IHR (2005) in Yemen. First, the interpretation of the data by reading each transcript and underlining statements. Second, all underlined statements were coded across each interview undergoing inductive analysis. Third, all codes were grouped into two themes: positive perceptions and negative perceptions. Finally, all statements in both themes were read to reflect the overarching key health informant’s perceptions about the implementation of the IHR (2005) in Yemen.

The results were tabulated, and technical areas in the resultant table were classified according to core functions “Prevent (P)”, “Detect (D)”, and “Response (R)”, as well as internationally related hazards (Chemical emergencies (CE), Radiological Emergencies (RE)) and Points of Entries (PoE). The qualitative data analysis was done by using key informant quotes to develop color scoring for the indicators of IHR (2005) core capacities based on the World Health Organization’s global JEE tool.

The JEE tool color scoring for the core capacity indicators is designed in the form of a five-number scale (1,2,3,4 and 5) , with specific color codes (Red, Yellow, and Green) given to each score. The detailed interpretation of the of the color-coded scoring system is as follows:

1. No capacity: Means there is no capacity in place for implementing IHR. Color Code: Red
2. Limited Capacity: The capacity is in its development stage (some tasks have been achieved, and some are in the process); overall, the country has started the process of implementation. Color Code: Yellow.
3. Developed capacity: The attributes of capacity are in Place, though, there is an obstacle in its sustainability due to various challenges (e.g. funding shortages). Color Code: Yellow.
4. Demonstrated Capacity: The attributes of the capacity are in place, though, it is sustainable for few more Years, and can be measures by IHR core capacity in the national health regulation plan. Color Code: Green
5. Sustainable Capacity: Core capacities are sustainable, functional, and the country is supporting other countries in implementing IHR, this is the highest level of achievement in the implementation of the IHR core capacities. Color Code: Green

In total, this study evaluated 49 indicators, and assigned color-coded scores in line with the pre-existing system acknowledged and in use by the WHO. Finally, the developed color scoring assessment was shared with three national IHR experts in Yemen and one international IHR expert for validation.

## Results

Table 1 (IHR Core Capacities Scoring in Yemen – 2019) shows the assessment results based on the World Health Organization’s JEE tool. Most indicators of the IHR core capacities scored less or equal to four. There are Seventeen out of forty-nine indicators were classified as no capacity, eighteen indicators were listed under the limited capacity, six indicators were listed as developed capacity, and eight indicators were classified as demonstrated capacity. No indicators were found to achieve sustained capacity.

**Table 1:**
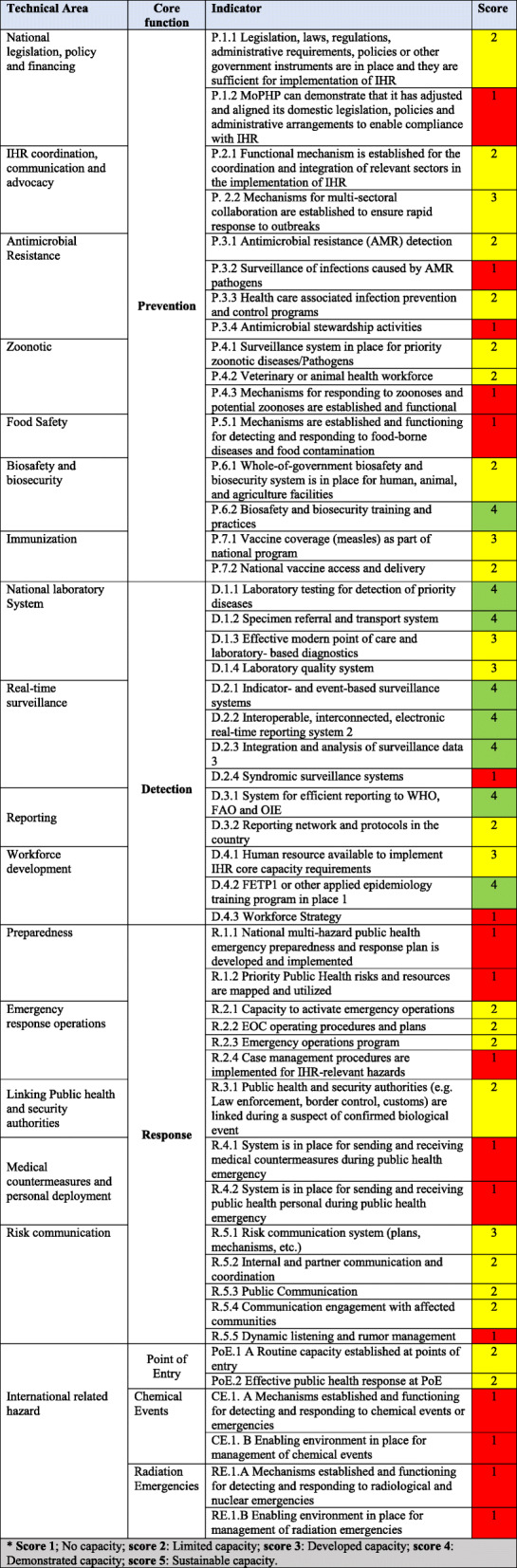
IHR Core Capacities Scoring - Yemen – 2019*

*Score 1; No capacity; **score 2**: Limited capacity; **score 3**: Developed capacity; **score 4**: Demonstrated capacity; **score 5**: Sustainable capacity

In the section below, we present the findings for the indicators under each of the three core functions of *prevention, detection, and response*.

### Prevention Core function indicators

There are sixteen prevention indicators in this study. Five of the indicators showed no capacity (for example, P.3.4 Antimicrobial stewardship activities), eight indicators showed limited capacity (for example, P.7.2 National vaccine access and delivery), two indicators showed developed capacity (P.7.1 Vaccine coverage (measles) as part of national program), and one indicator showed demonstrated capacity (P.6.2 Biosafety and biosecurity training and practices). According to the data extracted from the key informants:

“*There is an immunization program in the country at the governorate level, which was implemented in 1979 and is one of the oldest national programs in Yemen. It follows the policy of immunization through a university committee called the Immunization Technical Advisory Group on a regular basis, which organizes the standard of vaccination and has right to decide to publicly administer a new vaccine or not*”. **Key Informant 4.**

“ *… As I told you we do not do this training only for the field epidemiology training residents, but also we invited the main hospital and the infection control. We invited directors of PoE. We trained almost 200 people on biosafety and biosecurity, IHR, and outbreak investigations and we also involved the rapid response team in this training …* “. **Key Informant 9.**

### Detection Core function indicators

Overall, the detection core function showed high capacity. Only two indicators showed no capacity (for example: D.4.3 Workforce strategy), one indicator showed limited capacity (D.3.2 Reporting network and protocols in the country), three showed developed capacity (for example: D.1.4 Laboratory quality system), and seven out of the thirteen detection indicators showed demonstrated capacity (for example: D.2.3 Integration and analysis of surveillance data). According to the data extracted from the key informants:

“*Currently there is operationalized plan to extend the NCPHL to an additional four governorates in Yemen (Hajja, Sada’a, Amran and Al.Dhale) and the funds and human resources are in place[ …*]. *There is a serology department for Human Immunodeficiency Virus (HIV), Hepatitis B Virus (HBV), Hepatitis C Virus (HCV), Cytomegalovirus (CMV), Dengue Fever, measles, Rubella, Rotavirus and a microbiology department for microbiological and, biological samples, in addition to urine and stool culture. Regarding the poliovirus, we do not have the reagents*”. **Key Informant 10.**

“*There is an integrated committee between the NCPHL and Ministry of Public Health and Population specifically during the epidemics, and every week they conduct a meeting to share the data*”. **Key Informant 4.**

“*There is an electronic surveillance system for all the suspected cholera cases which is reported in the line list, and then a daily report is raised [ …*] “. **Key Informant 3.**

### Response Core function indicators

Overall, the response core function appeared to stand at a low capacity. Fourteen indicators were covered in this evaluation, out of which six indicators showed no capacity (for example: R.1.2 priority public health risks and resources are mapped and utilized), seven showed limited capacity (for example: R.2.1 capacity of active emergency operations), and one indicator showed developed capacity (R.5.1 Risk communication system (plans, mechanisms, etc.). Based on the reflections from the key informants:

“*The strategies for any health emergency in Yemen are based upon the occurrence of the crisis, without any previous plan in place. We are only distributing duties among the staff at the same time of outbreak (ad-hoc based)”*. **Key Informant 4.**

Two indicators of radiological emergencies (for example: enabling environment in place for management of radiation emergencies) and two indicators of chemical events (for example: enabling environment in place for management chemical events) showed no capacity, while two indicators of PoE showed limited capacity (for example: effective public health response at PoE).

## Discussion

**Fig. 1:**
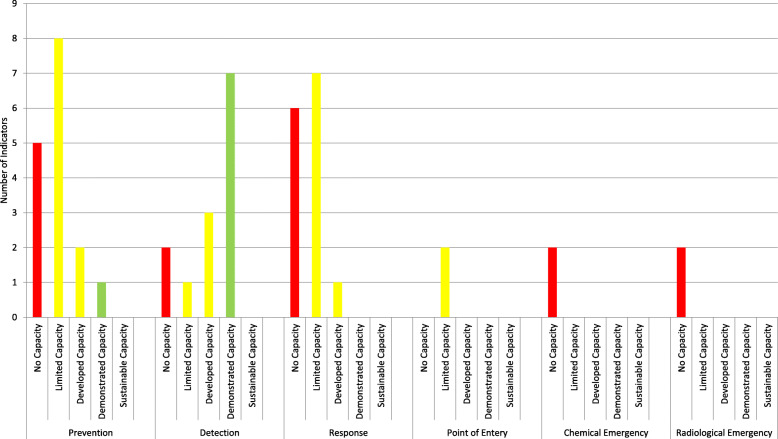
Implication of IHR Core Capacities Color Scoring - Yemen 2019

The findings of this study demonstrate an overall poor implementation of the IHR (2005) core capacities in Yemen. Even though Yemen has signed the agreement for IHR (2005) implementation as early as the year 2006, the country continues to face perpetual challenges in meeting its obligations under this crucial global health instrument of international law. Such situation is not unexpected in the case of Yemen. The ongoing conflict has affected various aspects of communication, coordination, and advocacy among the responsible national authorities, and between the national authorities on one hand and international agencies and NGOs on the other. The conflict also complicates a pre-existing disrupted health system, with a range of core health indicators at alarming rates over the decades. The results of the study show that Yemen has a higher capacity in the core function of detection, along with low capacity in the core functions of prevention and response see Fig. 1. As a consequence of the ongoing war in Yemen, the essential health infrastructure and the health system collapsed. This has led to aggravation of the spread of several communicable diseases including cholera, diphtheria, dengue hemorrhagic fever and COVID-19 [16]. It is important to highlight that the relatively high capacity to detect outbreaks does indeed fall short in the face of COVID-19 pandemic.

### Prevention Core function indicators

In addition to the above mentioned systemic challenges and complexities related to the collapse of essential service as a result of the war in Yemen, there has been inappropriate planning of resources and expenditure of funds for IHR (2005) implementation. In this context, the bureaucratic systems of the international community and the complex political situation present further challenges to any entity interested in supporting MoPHP to implement the IHR (2005) core capacities. This is especially dangerous when such complexity affects donor trust, readiness, and willingness to invest funds in Yemen. A study conducted in Tanzania suggested that insufficient budget allocation for IHR implementation is one of the main challenges to implementing IHR (2005) [17]. Yemen has qualified national public health professionals and academics working at MoPHP. These professionals are quite capable of developing the necessary guidelines for the prevention, detection, and response to various diseases of epidemic potential in the country. However, the financial support essential to the development and implementation of such guidelines and legislature is either non-existent or has not been allocated. Yemen is not the only low income, war affected country facing such situation. The findings of the current study are in line with similar findings of the JEE exercise conducted in Somalia in 2016 [18].

A view from the field shows the grim reality of systemic fragmentation and a proliferation of vertical programs imposed by INGOs in Yemen, further complicating the country’s capacity to implement the IHR (2005). INGOs come with their own sets of guidelines, protocols and strategies, and more often than not implement these sets in silos to achieve their agendas. Their interventions are often poorly aligned with the national strategies of MoPHP and frequently not well communicated on a regular basis to the authorities. This indicates complete disassociation between MoPHP and INGO agendas, and reflects the fragmentation in implementing the global health security agenda on the ground. INGO programs are mostly tailored to meet the reporting requirements of donor agencies, and do not see beyond the limited project cycle. Such modus operandi are counterproductive to the incremental progress towards achieving sustainability in the implementation of the IHR (2005) and the building of its core capacities at national level in Yemen and other low income, conflict affected countries. In Yemen, lack of orientation and awareness on the importance of IHR implementation among national and international partners is also evident, coupled with high rates of staff turnover and a vicious cycle of change in the personnel working with national health stakeholders and decision makers in the country.

On the global health scale, immunization represents a strong public health prevention program and has been estimated to prevent approximately two million global deaths per year [19]. However, national vaccine access and delivery in Yemen is still facing a variety of obstacles amidst the conflict. The results of this study revealed developed capacity in vaccination coverage and limited capacity in national vaccine access, similar to the case of Iraq [19].

### Detection Core function indicators

On the positive side, the “detection” core function indicators scored at the level of “developed capacity” reflect the best practices and strengths of the health system in Yemen in terms of implementing good tools to detect epidemics that are common in the region, such as cholera and diphtheria. For example, MoPHP uses an electronic Integrated Disease Early Warning System (eIDEWS), which is an important tool for the early detection of outbreaks [20]. Another important point related to the success of eIDEWS is implementing this system in the National Central Public Health Laboratories (NCPHL) for sharing the confirmed diagnoses of cholera cases and other epidemics. Having this tool within a collapsed health system is considered a great achievement in the midst of extended war in Yemen [21]. In contrast, the real time surveillance system in Somalia, is very weak and there is almost no capacity while in Iraq the surveillance system is between “limited capacity” to “developed capacity” [18, 22].

Furthermore, Yemen Field Epidemiology Training Program (FETP) continues to represent a strong applied program for workforce development since 2011 and up to date. Yemen FETP has played a key role in the detection of outbreaks through a system of outbreak investigation [23]. Several cohorts of field epidemiologists have graduated and filled the gap in field epidemiology in terms of investigation, data analysis and reporting to higher-level decision makers. This explains the continuous efforts towards strengthening detection core function in Yemen [23, 24]. Regarding to the point of entries, results showed limited capacity in Yemen, similar situation was noticed in Somalia and Iraq [18, 22].

### Response Core function indicators

The study has shown the lack of a well-established network for building the risk communication capacity, which falls under the response core function. There is no prioritization of risk communication to increase awareness about the danger of epidemic diseases. There is also no developed, formalized system for tracking rumors and misinformation, which is highly needed in the current context of Yemen particularly during the COVID-19 outbreak [1]. All of this exposes a population that is internally displaced and constantly on the move because of the war to multiplied risks from the lack of information, both on the potential risks, and on how to prevent and control the spread of outbreaks at community level. A similar finding in Iraq which showed almost identical scores to Yemen, reflecting the negative impact of political instability, war, and population displacement on the development of systems for rumor and misinformation tracking [22].

Despite the great efforts made by INGOs present in the country in the area of response to current public health crises, there is still weak coordination and ineffective communication between MoPHP and the relevant stakeholders. The development of a new cholera taskforce for response to the ongoing cholera outbreak in the country showed fluctuation and fragmentation in coordination and operationalization of the emergency plan, and the same was observed during the preparedness for COVID-19 [25].

The current study has identified several obstacles hindering the implementation of the risk communication IHR core capacity. These obstacles include the deteriorated communication infrastructure, difficult transportation, and lack of electricity to spread health education and awareness messages in Yemen. Furthermore, there is lack of administrative resources to ensure the planning, budgeting, and preparedness of risk communication activities, which should be in place well before an outbreak occurs [25].

Although the detection in Yemen demonstrated high capacity, the core capacities which are essential for prevention and response scored between no capacity and limited capacity, indicating weakness in prevention and response. Similarly, an obvious weakness in prevention was noticed in Iraq and Somalia [20, 23]. In the area of response, all three countries showed low capacity. The low response capacity found in Yemen, despite good detection capacity can be interpreted as an indicator of poor health financing, and lack of an overall response framework, both cornerstones to successful outbreak response at grassroots level.

## Conclusion

Taking all the evidence rendered by this study, demonstrating poor IHR (2005) implementation capacity in Yemen, this study recommends urgent measures to strengthen IHR implementation during the ongoing conflict. The recommendations include:

1. **Orientation:** dedicate significant effort to raising awareness among national health staff, and INGO personnel about the IHR framework and the importance of implementing it to achieve the agenda of global health security. The slogan of “no one is safe until we are all safe” repeatedly used by the Director General of the World Health Organization in relation to the COVID-19 pandemic is very relevant in this regard. Awareness and training on the IHR (2005) should be done tirelessly, to create a safety net in the face of the constantly changing health personnel and the high staff turnover.
2. **Alignment:** improve the alignment of INGO programs with those of the MoPHP and align the programs overall towards the effective implementation of the IHR (2005). This could be achieved through a multi-step, phased-approach plan, with clear milestones, concrete and SMART monitoring indicators, and realistic means of verification.
3. **Coordination:** establish and strive to implement an advanced level of coordination, networking, and transparency in terms of sharing epidemiological and other related data, and working together to develop, implement and monitor health security action plans.
4. **Funding:** ensure adequate funding, and targeted budgeting is allocated for all the areas of IHR (2005). The funds must trickle down to the level of activities and guarantee that awareness materials are not only developed but also disseminated effectively and creatively to all population categories in Yemen, with a special focus on Internally Displaced Persons (IDPs) and refugees.
5. **Innovative approaches:** incorporate up-to-date knowledge in analyzing disrupted health systems and develop approaches to bridge the divide between health system strengthening and emergency response.
6. **Addressing the root causes:** Finally, as this study identified that the ongoing conflict and political instability have strongly affected the health sector at different levels, it is crucial to address the root cause of all suffering by urgently resolving the protracted political crisis in Yemen. This would make space for effective rebuilding of the health system and re-establishment of modalities for the proper delivery of basic health services in Yemen.

## Supplementary Information

**Additional file 1.**

## Data Availability

The data was obtained from the IDI with the participants are available upon to request.

## Abbreviations

CE: Chemical emergencies
eIDEWS: Electronic integrated disease early warning system
FETP: Field epidemiology training program
IDI: In depth interviews
IDPs: Internally displaced persons
IHR: International health regulations
JEE: Joint external evaluation tool
MoPHP: Ministry of public health and population
NCPHL: National central public health laboratories
PHEIC: Public health emergency of international concern
PoE: Point of entries
RE: Radiological emergencies
SMART: Specific, measurable, achievable, realistic and time-bound

## References

[1] WHO. International Health Regulations. 2005. Avialable from https://www.who.int/health-topics/international-healthregulations#tab=tab_. (cited 2019, 10 July).

[2] Katz R, Standley CJ. Regional approaches for enhancing global health security. BMC Public Health. 2019;19:473. 10.1186/s12889-019-6789-y. 10.1186/s12889-019-6789-y PMC6696706 32326911

[3] Aldis W (2008). Health security as a public health concept: a critical analysis. Health Policy Plan.

[4] Menon AN, Rosenfeld E, Brush CA (2018). Law and the JEE: lessons for IHR implementation. Health Secur.

[5] Ijaz K, Kasowski E, Arthur RR, Angulo FJ, Dowell SF (2012). International health regulations—what gets measured gets done. Emerg Infect Dis.

[6] Philippe Calain, From the field side of the binoculars: a different view on global public health surveillance, Health Policy Plan, Volume 22, Issue 1, January 2007, Pages 13–20, https://doi.org/10.1093/heapol/czl035. 10.1093/heapol/czl035 17237490

[7] WHO. Development of a draft five-year global strategic plan to improve public health preparedness and response. 2017 [cited 2019 22 July]; Available from: https://www.google.de/url?sa=t&rct=j&q=&esrc=s&source=web&cd=2&ved=2ahUKEwiujMKUr-bjAhWLJFAKHbA3C3sQFjABegQIAhAC&url=http%3A%2F%2Fwww.wpro.who.int%2Fabout%2Fregional_committee%2F68%2Fdocuments%2Fwhe_cpi_ihr_5-year_global_strategic_plan.pdf&usg=AOvVaw0hVcocTWwNBY9zApdomlYJ.

[8] UNDP. Assessing the Impact of War on development of Yemen. 72 (2019).[Cited Aug, 2020]; Available from: https://www.undp.org/content/dam/yemen/General/Docs/ImpactOfWarOnDevelopmentInYemen.pdf

[9] El Bcheraoui C (2018). Health in Yemen: losing ground in war time. Glob Health.

[10] UNICEF. Evaluation of the UNICEF level 3 response to the cholera epidemic in Yemen: A crisis within a crisis. 2018 [cited 2019 3 July]; Available from: https://www.google.de/url?sa=t&rct=j&q=&esrc=s&source=web&cd=1&ved=2ahUKEwidjo2ikubjAhWIzqQKHe1hB94QFjAAegQIABAC&url=https%3A%2F%2Fwww.unicef.org%2Fevaldatabase%2Ffiles%2FEvaluation_of_UNICEFs_Response_to_the_Cholera_Outbreak_in_Yemen_EOHQ_2018-001.pdf&usg=AOvVaw3RR3iyrCHCMvP4s2menh-a.

[11] Alamodi AA, Eshaq AM, Fothan AM, Bakather AM, Obad AS (2015). Tackling preventable diseases in Yemen. Lancet.

[12] The Lancet. Yemen's silent killers. Lancet. 2017;389(10070). 10.1016/S0140-6736(17)30390-2 28229862

[13] Kuna A, Gajewski M (2017). Cholera - the new strike of an old foe. Int Marit Health.

[14] Kohlbacher Florian. The Use of Qualitative Content Analysis in Case Study Research [89 paragraphs]. Forum Qualitative Sozialforschung / Forum: Qualitative Social Research. 2005;7(1):21. 10.17169/fqs-7.1.75, http://nbn-resolving.de/urn:nbn:de:0114-fqs0601211.

[15] Kohlbacher F. The use of qualitative content analysis in case study research. In: Forum qualitative Sozialforschung/forum: qualitative social research: Institut für Qualitative Forschung; 2006.

[16] Dureab F, Shibib K, Al Yousufi R, Jahn A. Yemen: cholera outbreak and the ongoing armed conflict. J Infect Dev Ctries 2018; 12(5):397–403. https://doi:10.3855/jidc.10129 10.3855/jidc.10129 31865306

[17] WHO. Joint External Evaluation tool (JEE tool) first edition. 2016 [cited 2019 6 June]; Available from: https://www.who.int/ihr/publications/WHO_HSE_GCR_2016_2/en/.

[18] WHO. Joint External Evaluation of IHR Core Capacities of the Republic of Somalia. 2016 [cited 2019 28 July]; Available from: https://www.who.int/ihr/publications/WHO-WHE-CPI-2017.17/en/.

[19] Gaafar T, Moshni E, Lievano F (2003). The challenge of achieving measles elimination in the Eastern Mediterranean Region by 2010. J Infect Dis.

[20] Dureab F, Ismail O, Müller O, Jahn A (2019). Cholera outbreak in Yemen: timeliness of reporting and response in the National Electronic Disease Early Warning System. Acta Inform Med.

[21] Ahmed K, Altaf MD, Dureab F (2014). Electronic infectious disease surveillance system during humanitarian crises in Yemen. Online J Public Health Inform.

[22] WHO. Joint External Evaluation of IHR Related core capacities of Republic of Iraq. 2019 [cited 2019 28 July]; Available from: https://www.who.int/ihr/publications/WHO-WHE-CPI-2019.61/en/.

[23] Al Serouri A, Jumaan A, Alkohlani A (2018). Yemen field epidemiology training programme: a tool for strengthening the public health workforce. East Mediterr Health J.

[24] Yemen FETP. Y-FETP Launches its Fourth Cohort of Advanced Field Epidemiology Training. 2017 [cited 2019 18 July]; Available from: http://emphnet.net/?country_programs=yemen-fetp.

[25] Dureab F, Al Awlaqi S, Jahn A. COVID-19 in Yemen: preparedness measures in a fragile state. Lancet Public Health 2020; DOI: https://doi.org/10.1016/S2468-2667(20)30101-8, 5, 6, e311. 10.1016/S2468-2667(20)30101-8 PMC7180012 32334648

[26] World Medical Association. WMA Declaration of Helsinki – Ethical Principles for Medical Research Involving Human Subjects. 2013 [cited 2019 25 March]; Available from: https://www.wma.net/policies-post/wma-declaration-of-helsinki-ethical-principles-for-medical-research-involving-human-subjects/.

[27] WMA, World Medical Association Declaration Of Helsinki (2001). Ethical principles for medical research involving human subjects. Bull World Health Organ.

