# Correction to: Mind the gap: an analysis of core capacities of the international health regulations (2005) to respond to outbreaks in Yemen

**DOI:** 10.1186/s12913-021-06666-z

**Published:** 2021-07-09

**Authors:** Hanan Noman, Fekri Dureab, Iman Ahmed, Abdulwahed Al Serouri, Taha Hussein, Albrecht Jahn

**Affiliations:** 1 Heidelberg Institute of Global Health, Uniklinikum, Heidelberg, Germany; 2 Ruprecht-Karls-Universität Heidelberg, Heidelberg, Germany; 3 IRIA, Akkon-Hochschule für Humanwissenschaften, Berlin, Germany; 4 Independent Global Health Expert, Montreal, Canada; 5 Yemen Field Epidemiology Training Program, Ministry of Public Health and Population, Sana’a, Yemen; 6 Medicines Sans Frontiers –France, Aden, Yemen

**Correction to: BMC Health Serv Res 21, 477 (2021)**

**https://doi.org/10.1186/s12913-021-06395-3**

Following publication of the original article [1], a funding information was missing in the Funding section.

The updated Funding section is given below and the changes have been highlighted in **bold typeface**.

Funding

Open Access funding enabled and organized by Projekt DEAL. **This work was supported by the Wellcome Trust [grant number: 214686/Z/18/Z].**

The original article [1] has been corrected.
