# Supplementary material for: Mind the gap: an analysis of core capacities of the international health regulations (2005) to respond to outbreaks in Yemen
Source: BMC Health Serv Res. 2021 May 20;21:477. doi: 10.1186/s12913-021-06395-3 (PMC8134964; doi:10.1186/s12913-021-06395-3)
Supplement: Supplementary file 1 — Additional file 1. [file 12913_2021_6395_MOESM1_ESM.docx]

**Mind the Gap: An Analysis and conclusion on the International Health Regulations (2005) core capacities to respond to the outbreaks of infectious diseases in Yemen**

*In-depth interview with key informants at Ministry of Public Health and Population (MoPHP), Academia, and acting international organizations in Republic of Yemen*

| **Respondent information** | |
| --- | --- |
| Name of the Health Agency |  |
| Name/Sex of the respondent |  |
| Position |  |
| Respondent role at the health agency/MoPHP |  |
| Location |  |
| Date |  |
| Supported Facility |  |

**To identify the use and implication of the IHR in Yemen**

1. Describe the current Health Regulation core capacities in the Yemen? (identify the available national plans)

a- **Prevention**

Proxy: What are the administrative requirements that the country has identified to implement health regulation capacities?

- Protocols, Guidelines and Standard Operating Procedures (SOP) for Infection Prevention Control (IPC) programs.
- Availability of trained IPC professionals at local and intermediate level.
- Availability of system to evaluate the effectiveness of infection prevention measures regularly.
- Availability of Sustainable Immunization Program against epidemic prone Vaccine Preventable Diseases (VPDs) and having specific vaccination policy (e.g. targeting population at high-risk epidemic prone VPDs of national importance like Vibrio Cholera.

b- **Detection**

Proxy: What is the existing human resources capacity on epidemiologists, clinicians, biostatisticians, information systems specialists, laboratory technicians, and other public health personnel for the different health system levels (Local, intermediate, and national)?

- Availability of Surveillance System Team at national level who are able to produce reports and data to higher level of public health decision makers in the country and feedback to lower level implementing program.
- Availability of national laboratory policy which had linked to the surveillance system.
- Availability of national health regulation focal point at local level.
- Having strong linkage between national health regulation focal point and public health decision makers and other leadership in the country whenever there is identification of any public health event in the country.
- Availability of national public health workforce strategy.
- Including the existing administrative capacity of the human resources in the public health workforce.

**C**-**Response**

Proxy: What is the country emergency risk profile? Mechanisms for development and implementation of multi-hazard emergency preparedness. Give examples.

- Main core capacities of national emergency preparedness and response plane.
- Regular update of the plane if it is available.
- Availability of Emergency Operation Centre (EOC) at national level and its floor plan and description of its equipment's.
- Availability of trained Rapid Response Team at local, intermediate and district level.

**The implementation of IHR during preparedness for cholera outbreak in Yemen**

1. How does National Health regulation capacity developed, reviewed and operationalized in the country to response to the current cholera outbreak? (**Prevent, Detect, Response**)

Proxy: Is there an existing national plan for strengthening implementation of IHR capacities to control cholera outbreak (National Health Security, others) and has this plan been financed?

1. To what extent are these capacities being available to address cholera outbreak (at national level or below)? (**Prevent, detect, and respond**). (challenges)

Proxy: Describe the capacity that consumed for epidemiology, case management, laboratory at local and intermediate level? (Detect).

1. What are the preventive measurement taking place during the cholera outbreak? (Prevent)

Proxy: Dose immunization program activated against current cholera outbreak in high risk regions of the country? What is the target rate of vaccination campaign against cholera? What is the factors that depressed the vaccination campaign against cholera? (challenges).

1. What is the emergency preparedness for the current cholera outbreak which had already faced the country? (Response).
2. What are the measurement used to detect cholera cases? (Detect).

Proxy:

**A-Surveillance**

1-Dose the country utilize electronic surveillance system for the current cholera outbreak?

2-Are the data from this system shared with the health sectors in the country? How?

**B- Laboratory**

Describe the structure of laboratory system, number of laboratories? What is the cholera test that had been used in the country during the current cholera outbreak? How the laboratory data of positive cholera cases shared with the national surveillance system?

**To find out the stakeholders’ map in Yemen**

1. **Partners involvement and coordination:**
2. Could you identify the partners concern about the implementation of IHR in Yemen? And why?
3. How does the country ensure the coordination of legal and regulatory framework between sectors? (Show evidence, examples).

**To detect the challenges facing implementation of IHR**

1. What are the challenges facing IHR implementation in Yemen?
2. How can we overcome the obstacles and perform better to implement IHR during the conflict time, is there any recommendation or lessons to learn from Yemen?

**Thank you for your participation!**
